# Early Hepatocellular Carcinoma: Transplantation versus Resection: The Case for Liver Resection

**DOI:** 10.4061/2011/142085

**Published:** 2011-04-27

**Authors:** Nishant Merchant, Calvin S. David, Steven C. Cunningham

**Affiliations:** Department of Surgery, Saint Agnes Hospital, 900 Caton Avenue, Mailbox #207, Baltimore, MD 21229, USA

## Abstract

The optimal surgical treatment of hepatocellular carcinoma on well-compensated cirrhosis is controversial. Advocates of liver transplantation cite better long-term survival, lower risk of recurrence, and the ability of transplantation to treat both the HCC and the underlying liver cirrhosis. Transplantation, however, is not universally available to all appropriate-risk candidates because of a lack of sufficient organ donors and in addition suffers from the disadvantages of requiring a more complex pre- and postoperative management associated with risks of inaccessibility, noncompliance, and late complications. Resection, by contrast, is much more easily and widely available, avoids many of those risks, is by many accounts as effective at achieving similar long-term survival, and still allows for safe, subsequent liver transplantation in cases of recurrence. Here, arguments are made in favor of resection being easier, safer, simpler, and comparably effective in the treatment of HCC relative to transplantation, and therefore being the optimal initial treatment in cases of hepatocellular carcinoma on well-compensated cirrhosis.

## 1. Introduction

Hepatocellular carcinoma (HCC) is the seventh most common cancer worldwide, one of the most common causes of cancer death worldwide, and its incidence is increasing [1–3]. The rate of cancer death from primary liver cancer (90% of which is HCC [4]) in the United States has increased by over 40% in recent decades [2]. Risk factors for the development of HCC include hepatitis (most commonly hepatitis B virus (HBV) or hepatitis C virus (HCV)), steatohepatitis, cirrhosis, hepatotoxins, and less commonly hereditary diseases such as hemochromatosis and alpha-1 antitrypsin deficiency. HCC uncommonly arises in healthy liver parenchyma. HBV is the most common underlying liver disease, and chronic carriers have a logarithmically increased risk of developing HCC compared to the general population [4].

## 2. The Debate

There is currently no consensus regarding the best surgical treatment for patients with well-compensated cirrhosis and early HCC within the Milan criteria (a single tumor <5 cm in maximum diameter, or 2-3 tumors each <3 cm, without lymphovascular invasion [5, 6]). While transplantation is clearly better for patients with severe cirrhosis and early HCC, and resection is better than transplantation for resectable but extra-Milan-criteria HCC on mild cirrhosis, on the middle ground—early HCC with mild cirrhosis—wages the debate between transplantation and resection.

## 3. Advantages of Liver Transplantation

The ability to treat with a single intervention not only the HCC but also the underlying oncogenic liver disease from which it arose—and by extension, from which other tumors may arise—is one of the greatest advantages of liver transplantation over resection. In high-volume centers, liver transplantation achieves this goal with acceptable morbidity and mortality (Table 1).

Furthermore, not only is liver transplantation relatively safe, but compared with resection, it has been reported to produce a longer 5-year survival and a lower rate of recurrence (Table 1). The reasons for these improved results compared to resection are difficult to discern, however, and may be related to a truly superior extirpation of gross and microscopic disease or to selection bias, especially as might occur from inappropriate stratification based on stage of disease. Staging of HCC is in fact plagued by an inordinate number of staging systems. At the time of the recent American Hepato-Pancreato-Biliary Association/American Joint Commission on Cancer (AHPBA/AJCC) Consensus Conference on Multidisciplinary Treatment of HCC staging [7], there were 18 different staging or scoring systems—or versions thereof—in use around the world. A major reason that HCC staging is difficult is that, to a greater extent in HCC compared with other cancers, prognosis after surgical treatment of HCC depends not only on tumor factors, such as size, number, and invasiveness (as are used in AJCC staging), but also on factors related to patient comorbidities, performance status, and quality-of-life scores, factors related to liver disease, factors related to etiology of disease (e.g., alcohol versus hepatitis B versus hepatitis C), and interactions between these groups of factors [7]. Whatever the reason—selection bias or a true finding—the many available data suggest that the rates of long-term survival and recurrence after transplantation are superior to those observed following resection (Table 1).

In the early history of liver transplantation from the 1960s through the 1980s, transplantation was considered to be indicated for primary liver tumors not resectable by subtotal techniques [8–10]. However, recurrence rates as high as 82% [10] and single-digit 5-year survival rates [9] were disappointing. Subsequently, the observation [10–12] that small HCC identified on pathologic evaluation of explanted livers transplanted for other indications were associated with low recurrence rates and long-term survival led to the development of the above-mentioned Milan criteria. Patients meeting these criteria in the original study by Mazzaferro et al. had overall and recurrence-free 4-years survival rates of 85% and 92% percent, respectively, following liver transplantation [6]. These results have since been corroborated in subsequent series published in the 2000s, with recurrence rates as low as 2% and 5-year survival rates as high as 89% following liver transplantation for HCC (Table 1).

## 4. Advantages of Resection

### 4.1. Easier

Unfortunately, the high 5-year survival rates and the low recurrence rates possible following liver transplantation are available only to those patients waiting for a graft who actually get one, whereas resection is more easily and immediately available to all acceptable-risk patients. In fact, the national median waiting times based on Organ Procurement and Transplantation Network (OPTN) data as of December 2010 range from 140 days for American Indians to 651 days for Hispanics [13], during which time patients may drop out because of tumor or comorbid progression, death, or other reasons. Depending on the time period, type of analysis, and dropout criteria [14], the 1-year dropout rate for patients with HCC awaiting liver transplantation ranges from 12% to 38% [14–18]. When these dropouts were considered in one of the first intention-to-treat analyses [19], the 2-year survival decreased significantly from 84% to 54%. Although subsequent intention-to-treat studies accounting for dropouts have reported good 4-year survival rates of approximately 60% following transplantation for HCC [17, 18], other factors are not accounted for, such as socioeconomic barriers that may prevent many patients ever from being listed for transplantation. Resection, by contrast, is available more easily, widely, and immediately to all patients who can tolerate the operation. Not only is resection a modality that is easier for patients to obtain, but it is easier for surgeons to perform, since it almost never requires venovenous bypass and does not require transplantation fellowship training, which some but not all hepatobiliary fellowships include. Resection, however, should not necessarily be viewed as a mutually exclusive modality but rather a complementary one, since its easy availability makes it effective not only in achieving long-term survival, but also effective for use as both a selection tool for transplantation, and a bridge to transplantation, as discussed below.

### 4.2. Effective

Given the absence of randomized controlled trials comparing resection and transplantation, estimates of their relative effectiveness must at least be based on similar patient populations to avoid selection bias. To that end, several groups have studied transplantation-eligible patients, that is, patients meeting the Milan criteria for transplantation, who underwent resection, not transplantation. Such transplantation-eligible patients undergoing resection had 5-year survival rates of 70% at two large hepatobiliary centers [20, 21], a rate comparable with some of the best reported following liver transplantation (Table 1). In a more recent intention-to-treat analysis of 80 transplantation-eligible HCC patients who underwent resection compared to 293 patients listed for transplantation, 5-year survival was similar (66% and 58%, resp.) [22]. This is consistent with the observation in a 2009 review of nearly 60 series of resection and/or transplantation that the weighted mean of reported 5-year survival rates is similar for resection and transplantation: 48% and 52%, respectively [23]. 

Not only is resection effective at producing a 5-year survival comparable to that of transplantation, but in cases of recurrence—which is uniformly higher following resection compared with transplantation (Table 1)—transplantation remains an option. This strategy of salvage liver transplantation (SLT) has the advantage of limiting the impact on the available pool of donors since the majority of transplantation-eligible patients undergoing resection without recurrence would not draw from this valuable and limited resource of liver grafts.

Salvage transplantation was formally proposed first in 1998 by Llovet et al. [24], although several other authors were also studying this strategy around the same time [25–27]. Two simultaneously published articles in Annals of Surgery, by Adam et al. [28] and Belghiti el al. [29], popularized the approach in 2003, reporting disparate conclusions. Adam et al. compared 17 patients who underwent SLT for recurrence of HCC after resection with 195 patients following primary liver transplantation (PLT) and found significantly higher mortality (23.5%), shorter survival, and more recurrence in SLT patients compared to PLT [28]. Belghiti et al., by contrast, included an intention-to-treat analysis and found similar rates of complications, 5-year survival, and recurrence [29]. These latter results of Belghiti et al. have more recently been corroborated by other groups. Del Gaudio et al. reviewed the results of 227 cirrhotic patients with transplantation-eligible HCC: 80 who underwent liver resection and 147 liver transplantation [22]. Among the liver-resection patients, 49% recurred and of those who recurred, 69% were within the Milan criteria for transplantation, of whom 10 underwent SLT. Compared with patients who underwent primary transplantation, SLT patients had similar rates of complications, 5-year survival, and recurrence [22]. Cherqui et al. studied 67 transplantation-eligible patients who underwent resection and found that of 36 (54%) patients with a recurrence, 16 (44%) who underwent SLT had a 5-year survival rate of 70% [30]. 

*De principe* SLT is another strategy to minimize use of scarce liver grafts by using resection as a tool to select patients who, based on pathologic evaluation of the specimen, have risk factors for recurrence (e.g., microscopic vascular invasion, the presence of previously unrecognized small satellite nodules). The Barcelona Clinic Liver Cancer group has employed this strategy, finding it to be an effective way to improve the outcome of resected patients [31]. Of 17 patients who were candidates for either resection or transplantation, but who underwent resection, 8 were deemed high-risk and therefore offered immediate transplantation. Of 6 who agreed to *de prinicipe* SLT, 5 were transplanted and although 4 of these 5 had no pretransplantation evidence of HCC, 4 indeed were found to harbor unrecognized HCC in the explanted liver but were free of disease at a median follow up of 45 months [31]. 

The use of resection as an effective tool to select patients for *de prinicipe* SLT was corroborated by Scatton et al. who studied 93 patients who underwent curative-intent surgery for HCC, primary resection in 20 (all 20 of whom had well-compensated cirrhosis with a Model for End-Stage Liver Disease score of 8) and primary transplantation in 73 [32]. Six of the 20 resection patients underwent *de principe* SLT and 14 underwent SLT for actual recurrence. Not all 20 were within the Milan criteria at resection: Twelve (9 SLT and 3 *de principe*) were within and 8 (5 SLT and 3 *de principe*) were beyond the Milan criteria. The 20 patients undergoing resection followed by transplantation and the 73 undergoing PLT had 5-year survival rates (55% and 66%, resp.) that were statistically similar [32]. This study supports the notion that pathologic examination of resected specimens allows determination of which patients benefit most from an eventual transplantation, and allows the opportunity to perform it preemptively.

### 4.3. Safer

Not only is liver resection easier and as effective as primary transplantation, it is also likely safer. Although this claim is made with the understanding that there are no randomized controlled trials to support it, it is intuitively true, given that all transplantations are major and complex operations, even when done for small tumors. A liver resection for a small tumor, in a liver with well-compensated cirrhosis, however, is in general a lower-risk procedure, and can sometimes even be performed laparoscopically. In fact, a series of 163 liver resections for HCC (74% on cirrhosis) performed at 3 large European centers recently reported median operative time of 180 min, blood loss of 250 mL, and tumor size 3.6 cm, with a mean length of stay of 7 days [33]. A recent review of nearly 60 series of either transplantation, resection, or direct comparisons of the two modalities in the treatment of early HCC found that the weighted means of postoperative morbidity rates was nearly identical (44% for resection and 45% for transplantation), but mortality following transplantation was 60% higher than following resection (8% and 5%, resp.) [23]. 

While both resection and transplantation may be performed safely, resection has the additional advantage of delaying need for and risks associated with immunosuppression. These risks include toxicities (especially nephrotoxicity), infectious complications, and posttransplantation *de novo* neoplasms, among others. Nephrotoxicity is common after liver transplantation and adversely affects graft and patient survival [34]. Immunosuppression-related posttransplantation infection is a significant problem that is entirely avoided with resection. In a series of 1000 liver transplantations treated with tacrolilmus immunosuppression, posttransplantation infection was the most common cause of death (34% of 360 deaths) [35]. In cases of HCV-related HCC, reinfection of a new liver graft following transplantation is universal and serum HCV levels have been shown to increase 4- to 100-fold during treatment for acute rejection [36]. Posttransplantation neoplasms occur at a rate several-fold higher than age- and sex-matched individuals [37], and include skin cancers and lymphoma (up to 10-fold risk) [37–40], myelodysplastic syndrome [41], and other extrahepatic cancers, such as those of the head and neck, lung, and gastrointestinal tract [42].

### 4.4. Simpler

In addition to being safer, easier, and comparably effective relative to transplantation, resection has the advantage of simpler preoperative and postoperative management. Any patient being evaluated for either modality requires extensive workup regarding HCC and comorbid factors, but transplantation requires in addition an extensive preoperative process that includes myriad wait-list issues, psychosocial evaluation of recipients and live donors, and the universal emergent nature of the operations. 

Bryce et al. [43] have studied the impact of sociodemographic factors on access to transplantation services and identified six stages that a patient must pass through prior to transplantation: disease occurrence, disease progression, disease diagnosis, referral for transplantation, listing for transplantation, and finally organ transplantation. Reasons preventing patients from completing all of these stages are numerous and include medical unsuitability for a transplantation, refusal of treatment, disparities/bias, and death. Using Pennsylvania state databases to collect sociodemographic and socioeconomic information, they linked data to records from five centers responsible for 95% of liver transplantations in Pennsylvania, and found that patients were significantly less likely to undergo evaluation, wait-listing, and transplantation if they were women, African American, or lacked commercial insurance [43]. Furthermore, these differences were greater during the early stages of the preoperative process (referral and listing) than for the final transplantation stage, where national oversight and review occur [43].

Postoperative management is similarly complex and requires a higher level of dedication, compliance, and investment of time, energy, and attention on the part of the patient than is possible for many patients, especially those of lower socioeconomic status. Noncompliance with immunosuppressive regimens and follow-up schedules has obvious risk for graft rejection and systemic toxicity and is more common in patients of low socioeconomic status [44]. Furthermore, for reasons that are not well defined, low-socioeconomic patients may also have worse survival following transplantation for HCC. In a study of 4735 patients identified in the OPTN database, although the survival of all patients with HCC improved *over time* regardless of racial, ethnic, and income groups, African American and low-income individuals had significantly poorer long-term survival compared to other socioeconomic groups [45].

## 5. Conclusion

Although liver transplantation provides the best recurrence-free survival and the best chance for a cure of HCC on well-compensated cirrhosis, due to the complete removal of all hepatic HCC disease and all oncogenic cirrhotic liver, the current (and likely future) shortage of available grafts, and the increased risks and complexities associated with the pre-, intra-, and postoperative course of liver transplantation counterbalance this advantage of transplantation. Furthermore, in cases of recurrence (or high risk thereof)—the one clear disadvantage of resection—transplantation remains a safe option. Taken together, these arguments suggest that resection is easier, safer, simpler, and as effective compared with transplantation and therefore is the optimal first choice for patients with early HCC on well-compensated cirrhosis.

## Figures and Tables

**Table 1:** Series comparing RSX and LT for HCC on cirrhosis.

First author [ref]	Year	N RSX	N LT	Mb (%) RSX	Mb (%) LT	Mt (%) RSX	Mt (%) LT	Overall 5-YS RSX	Overall 5-YS LT	Rec (%) RSX	Rec (%) LT
Iwatsuki^wi/o^ [46]	1991	17**	71**	NR	NR	NR	NR	0%	41%	50	43
Ringe^wi/o^ [47]	1991	131**	61**	NR	NR	15^30-d,∗^	15^30-d,∗^	36%	15%	NR	NR
Vargas^wi^ [48]	1995	35	11	NR	NR	NR	NR	58% 1-YS	81% 1-YS	40	0
Tan^wi/o^ [49]	1995	12	15	33^NR^	13^NR^	8.3^NR^	6.7^NR^	33% 3-YS	63% 3-YS	45	15
Michel^wi/o^ [50]	1997	102	113	39^NR^	38^NR^	8.8^NR^	22^NR^	31%	32%	86	30
Philosophe^wi/o^ [51]	1998	67**	58**	NR	NR	44^30-d^	13^30-d^	38%	45%	55	20
Colella^wi/o^ [52]	1998	41	55	NR	NR	NR	NR	44%	68%	NR	NR
Mazziotti^NR^ [53]	1998	238	41	42^NR^	80^NR^	4.6^30-d^	6.2^30-d^	41%	69%	NR	NR
Otto^wi/o^ [54]	1998	52	50	NR	NR	21^30-d^	8.0^30-d^	37%	44%	21	8.0
Weimann^NR^ [55]	1999	32	31	NR	NR	13^30-d^	10^30-d^	34%	63%	19	0
Yamamoto^wi/o^ [25]	1999	294	270	NR	NR	1.4^30-d^	7.8^30-d^	47%	54%	NR	NR
Llovet^wi^ [19]	1999	77	87	NR	NR	3.9^90-d^	2.3^90-d^	51%	69%	57	3.4
Figueras^wi/o^ [56]	2000	35	85	NR	6.7^NR^	NR	NR	51%	60%	65	7.0
De Carlis^wi/o^ [57]	2001	131	91	NR	NR	4.5^90-d^	18^90-d^	38%	65%	62	7.0
Shabahang^NR^ [58]	2002	44	65	NR	NR	7.0^NR^	7.0^NR^	57% 3-YS	66% 3-YS	NR	NR
Bigourdan^wi^ [59]	2003	20	17	30^30-d^	47^30-d^	5.0^30-d^	0^30-d^	36%	71%	30	18
Pierie^NR^ [60]	2005	81	33***	NR	NR	20^30-d^	9.0^30-d^	10%	19%	NR	NR
Margarit^wi^ [61]	2005	37	36	NR	NR	2.7^30-d^	5.6^30-d^	78%	50%	59	11
Poon^wi^ [62]	2007	204	43	35^30-d^	44^30-d^	3.4^H^	0^H^	68%	81%	NR	NR
Cillo^wi/o^ [63]	2007	131	40	NR	NR	5.3^90-d^	7.5^90-d^	31%	63%	53	5.0
Del Gaudio^wi^ [22]	2008	80	293	NR	79^NR^	0^NR^	5.0^NR^	66%	58%	59	46
Bellavance^wi^ [64]	2008	245	134	49^30-d^	65^30-d^	1.6^30-d^	1.5^30-d^	46%	66%	50	14
Sotiropoulo^wi/o^ [65]	2009	61	60	38^30-d^	38^30-d^	23^30-d^	8.0^30-d^	23%	59%	NR	NR
Zhou^wi^ [66]	2010	1018	89	NR	NR	0.69^NR^	4.5^NR^	70%	89%	NR	NR

Abbreviations: RSX: resection; LT: liver transplantation; YS: year-survival; Mb: morbidity; MT: mortality; Rec: recurrence.

*RSX and LT combined.

**Cirrhotic and noncirrhotic livers combined.

***33 wait-list patients (22 transplanted patients).

^wi^All patients within Milan criteria.

^wi/o^Some patients within and some outside of Milan criteria.

^NR^Milan criteria not reported.

^H^Hospital mortality (during same admission for same treatment).
